# Epidemiology of Carbapenem-Resistant *Enterobacteriaceae* Bacteremia in Gyeonggi Province, Republic of Korea, between 2018 and 2021

**DOI:** 10.3390/antibiotics12081286

**Published:** 2023-08-04

**Authors:** Seung Hye Lee, Chan Hee Kim, Hee Young Lee, Kun Hee Park, Su Ha Han

**Affiliations:** 1 Gyeonggi Infectious Disease Control Center, Health Bureau, Gyeonggi Provincial Government, Suwon-si 16508, Gyeonggi-do, Republic of Korea; lshbbb1208@gg.go.kr (S.H.L.);; 2 Center for Preventive Medicine and Public Health, Seoul National University Bundang Hospital, Seongnam-si 13620, Gyeonggi-do, Republic of Korea; ehy13@snubh.org; 3 Pyeongchang County Health and Medical Center, Pyeongchang-gun 25374, Gangwon-do, Republic of Korea; srhealth@korea.kr; 4 Department of Nursing, College of Medicine, SoonChunHyang University, Cheonan-si 31151, Chungcheongnam-do, Republic of Korea

**Keywords:** carbapenem-resistant *Enterobacteriaceae* (CRE), carbapenemase-producing *Enterobacteriaceae* (CPE), non-survivors, epidemiologic characteristics

## Abstract

The incidence of carbapenem-resistant *Enterobacteriaceae* (CRE) has been increasing since 2008, with Gyeonggi Province in South Korea being particularly vulnerable due to its large number of healthcare facilities. This study examines the trends of CRE occurrence in Gyeonggi Province over the past four years and the epidemiological characteristics of the infected patients. Patients with positive CRE blood cultures admitted to healthcare facilities in Gyeonggi Province from January 2018 to December 2021 were evaluated in this study. Risk factors for CRE-related death were analyzed using data from patients who died within 30 days of the last blood sampling. Older adults aged 70 years and above constituted the majority of patients with CRE bacteremia. Antibiotic use did not significantly affect mortality risk. Non-survivors were more common in tertiary hospitals and intensive care units and included patients with hypertension, malignant tumors, and multiple underlying diseases. *Klebsiella pneumoniae* was the most common CRE strain, with *Klebsiella pneumoniae* carbapenemase being the predominant carbapenemase. Our study suggests the endemicity of CRE in Gyeonggi Province and highlights the increasing isolation of CRE strains in South Korean long-term care hospitals within the province. Further, infection control measures and government support specific to each healthcare facility type are crucial.

## 1. Introduction

The increasing prevalence of infections caused by bacteria that produce carbapenemases has been recognized as a global public health threat [1,2]. While carbapenems are considered the “last resort” for the treatment of multidrug-resistant Gram-negative bacterial infections, the growing incidence of antimicrobial resistance has necessitated a greater use of carbapenems, thereby increasing the prevalence of carbapenem-resistant *Enterobacteriaceae* (CRE) [3,4]. Over the past 4 years, 45,436 cases of CRE infection have been reported in South Korea, and the number of reported cases and reporting in healthcare facilities has continuously increased [3]. In South Korea, since the first report in 2008, the frequency of CRE infections has increased rapidly every year. In December 2010, CRE infection was designated as a legal infectious disease and controlled using a sentinel surveillance system. The domestic outbreak in 2015–2016 was followed by a switch to a mandatory surveillance system in June 2017 for monitoring, patient management, and outbreak response [5]. With the recent increase in reports of CRE epidemics in healthcare facilities and the extremely high incidence and fatality rates [6,7,8,9], especially in CRE epidemics in long-term care facilities wherein the mortality rate is higher than in other healthcare facilities, the importance of CRE is being further emphasized [7,10].

Since CRE is transmitted through direct or indirect contact with infected patients, pathogen carriers, or through contaminated instruments, items, or environmental surfaces, infection control in healthcare facilities is crucial for disease prevention [11,12]. Although the scope and intensity of recommendations for the prevention and management of CRE epidemics are diverse, early detection of CRE and active CRE monitoring is recommended for high-risk groups showing risk factors for transmission during hospitalization [11,12,13]. Screening is also recommended before or during hospitalization in healthcare facilities [13].

The risk factors for CRE infection include previously identified CRE colonization/infection, utilization of a urinary catheter, and the administration of broad-spectrum anti-Gram-negative drugs [14]. In addition, the administration of antibiotics, patients’ comorbidities, and factors related to hospital settings have been identified as significant determinants associated with mortality in patients diagnosed with CRE infections [15].

Most patients with CRE infections are simple carriers and do not receive treatment; however, many strains of bacteria show resistance to various classes of antibiotics, making clinical treatment difficult. CRE mainly causes urinary tract infections as well as various infections such as gastroenteritis, pneumonia, and sepsis. In addition, it affects patient prognosis and is associated with a high mortality rate of 40–50% [16].

According to the national healthcare facility standard data (as of October 2022) obtained from facilities that can accommodate more than 30 patients, Gyeonggi-do contains the largest number of healthcare facilities in South Korea (877/4644, 18%), including 328 of the 1525 long-term care facilities nationwide, highlighting the regional characteristics that make it more vulnerable to CRE infections, thus requiring major management. Therefore, in this study, we analyzed the cases of CRE bacteremia between January 2018 and December 2021 in Gyeonggi-do and examined the factors affecting CRE-related deaths in these cases. These findings are expected to clarify the epidemiological characteristics of CRE-related deaths and present the basis for countermeasures against infectious diseases and managing CRE infections in South Korea.

## 2. Results

### 2.1. Demographic and Clinical Characteristics

Table 1 presents data on the sex, age, hospitalization history, and healthcare institutions of patients with CRE bacteremia from 2018 to 2021, classified by survivors and non-survivors. Among the 454 reported cases in which CRE strains were isolated from blood cultures, 57 deaths were noted. The survivors included 216 males and 181 females. The median age of the non-survivors and survivors was 72 (range, 50–97) and 71 years (range, 0–96) years, respectively. Among the survivors was an infant aged 0 years. In comparisons based on age, the number of deaths was higher in the age groups of 30s, 40s, 50s, and 90s; the number of survivors was higher in the age groups of 60s, 70s, and 80s. Although CRE was occasionally identified during outpatient or home nursing care, survivors’ hospitalization rates were higher than non-survivors (92.2% for survivors and 80.7% for deaths). In contrast, the intensive care unit (ICU) admission rate was higher among non-survivor cases (26.3% in deceased cases and 25.4% in survivors). Additionally, the percentage of cases confirmed through outpatient visits and home nursing was higher among deceased cases (7.0% for deceased cases and 4.5% for survivors). In analyses based on the type of healthcare facilities, CRE reports were received from tertiary hospitals, general hospitals, long-term care facilities, and hospitals. Tertiary hospitals accounted for the highest proportion of deaths (27/57 deaths, 47.4%), followed by general hospitals (25 deaths, 43.9%), long-term care facilities (4 deaths, 7.0%), and hospitals (1 death, 1.7%). When examining the distribution of deaths by type of healthcare facility and year, the percentage of reported cases from general hospitals was the highest in all 4 years, while the number of reported cases in tertiary hospitals was high in 2020 but decreased in 2021. Long-term care facilities and hospitals showed the same pattern in 2019 and 2020 but with an increasing trend in 2021. The proportion of survivors was the highest in general hospitals (211/397 cases, 53.1%), followed by tertiary hospitals (146 cases, 36.8%), long-term care facilities (32 cases, 8.1%), and hospitals (8 cases, 2.0%) (Table 1). 

**Figure 1:**
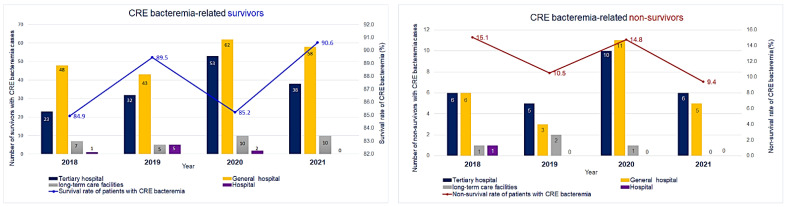
Carbapenem-resistant *Enterobacteriaceae* bacteremia-related survivors and non-survivors by healthcare facility type, 2018–2021.

The distribution of CRE bacteremia-related survivors and non-survivors in healthcare facilities in Gyeonggi-do reveals that the number of survivors in hospitals gradually decreased in 2020. In long-term care facilities, the number of survivors increased in 2020 and showed the same trend in 2021; in general hospitals and tertiary hospitals, the number of survivors increased in 2020 and then decreased again from 2021 onward. No CRE-related deaths were reported from hospitals in 2019, 2020, and 2021 and from long-term care facilities in 2021. In general hospitals and tertiary hospitals, the number of non-survivors increased in 2020 with a similar trend in the number of survivors but decreased again in 2021 (Figure 1). Antibiotic usage was higher among survivors; a history of antibiotic usage was reported for 40.6% of the survivors and 29.8% of the non-survivors. However, mortality risk did not differ significantly with the usage of antibiotics.

Further, information on underlying diseases was collected only for those who tested positive for carbapenemase-producing *Enterobacteriaceae* (CPE); thus, among 454 cases, this information was obtained only for 214 patients reported as showing positive results for CPE (Table 2). 

The underlying diseases identified in the CPE infection report were primarily categorized as follows: diabetes, stroke, renal failure, dialysis, chronic obstructive pulmonary disease, immunosuppressed patients, liver disease, and cancer, while other underlying diseases were categorized as others. Among the 214 patients whose underlying disease information was collected, underlying diseases were more frequently found among deceased patients than in survivors (186/397 [46.9%] survivors vs. 28/57 [49.1%] non-survivors). Among the eight underlying diseases, diabetes accounted for the highest proportion of survivors (35.5%, 66 cases), followed by malignant tumors (29.0%, 54 cases) and hypertension (26.9%, 50 cases). In contrast, malignant tumors were the most frequent underlying diseases in non-survivors (57.1%), followed by diabetes (32.1%, 9 cases) and stroke (14.3%, 4 cases). Except for hypertension and malignancy, none of the other underlying diseases showed differences in incidence between survivors and non-survivors. In our analysis of the risk factors for death in relation to underlying disease with the odds ratio (OR), hypertension showed an OR of 4.83 (95% confidence interval, 1.045–22.328), indicating that the odds of death in patients with hypertension were 4.83 times higher than that in those without hypertension. 

### 2.2. CRE Occurrence Distribution by Strains and Digestive Enzymes

From 2018 to 2021, a total of 454 cases of CRE bacteremia were reported in all 43 public health centers in Gyeonggi-do (Table 3). 

After excluding 15 cases in which the causative strains were not reported, *Klebsiella pneumoniae* comprised the largest proportion in both survivors (230 cases, 59.6%) and deceased patients (37 cases, 69.8%). A total of 241 cases (53.1%) were CPE-positive. Of the 213 cases in which CPE-related information was not inputted, in 18 cases, the confirmation test was not performed due to negative CPE results, whereas, in 10 cases, it was due to input errors. Of the 29 deaths among the 241 CPE-positive cases, 25 (86.2%) showed *Klebsiella pneumoniae* carbapenemase (KPC) and 5 (17.2%) showed New Delhi metallo-β-lactamase (NDM) with respect to the analyses of degradation enzymes. Among the survivors, 151 cases (71.2%) showed KPC, 49 (23.1%) showed NDM, and 11 (5.2%) showed oxacillinase (OXA) in the same pattern among the non-survivors (Table 4). 

**Figure 2:**
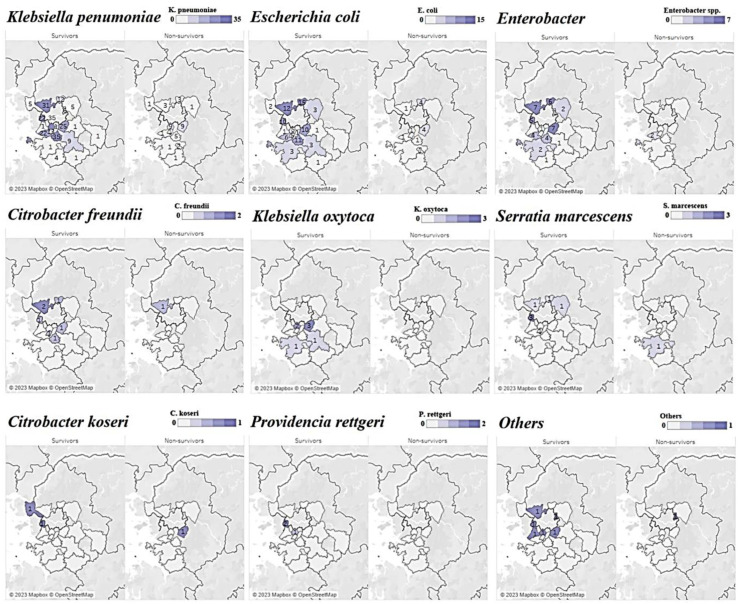
Choropleth map of carbapenem-resistant *Enterobacteriaceae* bacteremia in Gyeonggi Province, South Korea, 2018–2021.Note: The numbers on the top-right purple gradation scale indicate the cases of each strain of CPE.

The distribution of occurrence by region for each strain over 4 years showed similar patterns in the survivor and non-survivor groups, and *K. pneumoniae*, *Escherichia coli*, and *Enterobacter* strains were identified in areas where general hospitals and tertiary healthcare facilities were concentrated (Figure 2).

## 3. Discussion

In our analysis of CRE bacteremia reported from 2018 to 2021 at healthcare facilities in the Gyeonggi-do province, patients aged ≥70 years comprised the largest proportions of both survivors and non-survivors. Since most patients were confirmed to be inpatients, hospitalization history was a risk factor for CRE-related mortality (Table 1). Further, since the need for critical care treatment and the possibility of hospitalization increased with age, these findings were consistent with the results of several previous studies in which the risk factors for acquiring antibiotic-resistant bacteria included a history of hospitalization in an ICU and a prolonged hospitalization period [16]. 

In analyses based on the type of healthcare facility, the proportion of survivors was high in secondary hospitals, but the proportion of non-survivors was the highest in tertiary hospitals (Table 1). These results can be interpreted in two ways. First, when critically ill patients were transferred from long-term care facilities and secondary hospitals to tertiary hospitals, CRE carriers were identified in a pre-hospitalization screening and classified as external infections in some cases, thereby being excluded from reporting. Second, some cases were related to risk factors for CRE colonization, such as invasive treatment and a long-term ICU stay [17]. Since critically ill patients account for a larger proportion of the cases in tertiary hospitals than in secondary hospitals and long-term care facilities, they are more likely to undergo invasive procedures, such as mechanical ventilation and catheter insertion, which are known risk factors for death due to CRE infection. Although a history of antibiotic use was not a significant risk factor for death, 40.6% of survivors had a history of antibiotic use, suggesting that antibiotic use may emerge as a risk factor for mortality in the future. In previous studies, the use of antibiotics such as cephalosporins, β-lactams or β-lactam/β-lactamase inhibitors, aminoglycosides, fluoroquinolones, and glycopeptides was reported as a risk factor for CRE colonization [18,19,20,21,22].

In our analysis of underlying diseases (Table 2), diabetes was the most frequent underlying disease among survivors (66 cases, 35.5%). Consistent with the results of this study, diabetes is known to increase the risk of bloodstream infection as well as CRE infections [19,23]. In contrast, malignant tumors were the most frequent underlying diseases among non-survivors (16 cases, 57.1%). Although these results were not consistent with those of a previous study [20]—in which underlying diseases related to immunosuppressed conditions, such as malignant tumors and the use of immunosuppressive drugs were not risk factors for death—among the underlying diseases in the non-survivors, hypertension and malignant tumors appeared to be correlated with survival. The odds of death in patients with hypertension were 4.83 times higher than those without hypertension (Table 1). Hypertension is a chronic disease that often accompanies long-term hospitalization, and patients with malignant tumors require long-term hospitalization, suggesting that exposure to antibiotics and frequent admission to healthcare institutions may be risk factors for CRE colonization. 

In our analyses of the incidence trends by strain and region (Figure 2), *K. pneumoniae* accounted for the largest proportion regardless of patient survival, suggesting that endemicization had already progressed in the Gyeonggi-do area. As for *E. coli* and *Enterobacter*, which accounted for the second-largest proportion after *K. pneumoniae*, the range of cities and counties where CRE was reported showed gradual expansion. For strains such as *Citrobacter koseri*, *Providencia rettgeri*, *Serratia marcescens*, and *Raoultella ornithinolytica*, which have been reported in fewer than 10 cases (in Gyeonggi-do, as of 2022), no investigation has been conducted in relation to outbreaks, and further investigation of individual infection routes in relevant cases may reveal epidemiological links. In evaluations of the history of exposure to antibiotics and the history of visits to healthcare facilities, which are known to increase the risk of CRE infection, the occurrence of strains was the highest in densely populated areas and in areas where tertiary hospitals and general hospitals were widely distributed. 

In analyses based on carbapenemase, KPC accounted for the largest proportion (Table 4), showing the same pattern as the overall CRE and CPE separation trend in South Korea [5]. The findings of overseas CRE surveillance studies indicate that *K. pneumoniae* is one of the most commonly reported CRE strains worldwide [24,25,26,27]. As for degrading enzymes, KPC-producing bacteria has been reported to be endemic in a large number of countries [25,26,27,28,29]. The incidence trend and disease burden indicate that KPC endemicization has already progressed in the Gyeonggi-do province. 

Despite yielding a number of meaningful results, this study was subject to several limitations. First, when analyzing the underlying disease, information on other non-parameterized chronic diseases, such as hypertension and notably, pneumonia and urinary tract infections related to CRE, were also included. Unlike other variables whose input methods were standardized, this information was entered in the form of a memo, making it difficult to use for analysis. These elements, important as they are, could not be thoroughly analyzed because they were not required elements in the statutorily mandated infectious disease report form. For more scientific disease surveillance activities, the patient’s medical information should be collected as accurately as possible through a more systematic revision and supplementation of case reports, given that CRE is a healthcare-associated infection. Second, the structure of the case report form allowed for the entry of hospitalization and sample collection dates; however, the discharge date could not be confirmed. Moreover, only the past in-hospital movement route was entered if the patient was in the hospital on the reporting date. Since the duration of hospitalization is a well-known risk factor for CRE/CPE acquisition, it is regrettable that its relationship could not be confirmed through actual data. Third, the management of healthcare-associated infectious diseases guidelines of the Korea Disease Control and Prevention Agency (KDCA) advise reporting CRE and CPE infections only when strains and enzymes are added or changed or when CRE is confirmed in the blood after it is isolated from clinical samples other than blood. If the classification of the patient as a carrier of the pathogen is maintained when CRE is confirmed in clinical samples other than a blood sample, after being first isolated from blood, additional notification is not required. Therefore, when CRE is isolated from samples such as sputum or urine, the possibility that even fecal (including rectal swab) samples have been actually collected by healthcare institutions but not reported cannot be excluded. Fourth, according to current guidelines, cases involving CRE isolated from feces, sputum, or samples other than blood are not reported to the KDCA reporting system; therefore, the actual number of non-survivors in healthcare facilities may be underestimated. Future reports should consider including samples other than blood to provide data that could offer a closer analysis of the risk factors for death. Fifth, while the correct use of antibiotics is an important strategy for preventing multidrug-resistant bacterial infections, confirming the exact duration of antibiotic administration was challenging, as only the presence and type of antibiotics administered within 3 months could be entered in the case report. Our team of researchers aimed to conduct both multivariate and univariate analyses pertaining to risk factors associated with the correct use of antibiotics and source of infection. However, due to the current format of the national legally mandated infectious disease reports and the case investigation forms, there are numerous variables, including overlapping underlying conditions, complicating the performance of analyses to ascertain direct associations.

Despite these limitations, this study is meaningful in that it identified changes in the CRE occurrence trends, and the epidemiological characteristics related to the deaths of patients showing blood cultures positive for CRE/CPE based on 4-year cumulative reporting data. Since the CRE pattern change in South Korea was predicted several years ago, this study confirmed the previous results [4], suggesting CRE pattern changes and identified hospitalization history and underlying diseases as characteristics of CRE-related deaths.

For high-risk patients who are vulnerable to CRE infection due to the frequent use of antibiotics and use of multiple invasive devices in acute care hospitals, the Gyeonggi-do Center for Infectious Disease Control and Prevention conducts CRE testing upon admission to long-term care facilities [8] and enforces preemptive contact precautions before reporting the results. According to the World Health Organization guidelines [30], intensive training programs are being conducted for long-term care facility managers and infection control officials in the Gyeonggi-do province, including continuous recommendations for antibiotic use management, isolation and infection monitoring, and environmental management.

The number of CRE reports nationwide has been increasing every year, even during the coronavirus disease 2019 (COVID-19) pandemic [5,7,21]. Despite the policy to strengthen infection control in healthcare institutions during the pandemic, CRE infection cases in the long-term care facilities and hospitals in the Gyeonggi-do province increased in 2021 in this study. Thus, while struggling to respond to COVID-19, infection control officials may have also found it difficult to regularly monitor infection control in the hospital and to focus on managing multidrug-resistant bacteria.

The COVID-19 pandemic has changed the healthcare system and, at least temporarily, increased the rates of healthcare-associated infections and multidrug-resistant bacteria worldwide [31,32]. A study investigating healthcare-associated infections during the COVID-19 epidemic in 53 hospitals in the United States showed a significant increase in the rates of central line-associated bloodstream infection, ventilator-associated events, and *Clostridioides difficile* infections in small community hospitals. In this regard, establishing a cooperative system involving the government, regional institutions, and medical systems, focused on understanding and exploring the problems encountered by infectious disease specialists and the resource constraints faced by community hospitals, is suggested [33]. Although the COVID-19 epidemic has passed the critical stage, maintenance of a sustainable policy to prevent healthcare-associated infections and multidrug-resistant bacteria against new infectious diseases occurring in the future; infection control infrastructure suitable for each type of medical institution should be established; and the government’s support measures should be differentiated accordingly.

This study examined the epidemiological characteristics of CRE incidence and CRE bacteremia-related survivors and non-survivors from CRE-blood-culture-positive patients admitted to medical institutions in Gyeonggi-do from January 2018 to December 2021. The majority of survivors and non-survivors with CRE bacteremia were older than 70 years of age. Non-survivors were more common in tertiary hospitals and intensive care units and included patients with hypertension, malignant tumors, and multiple underlying diseases. Antibiotic use did not significantly affect mortality risk. *K. pneumoniae* was the most common CRE strain, and the predominant carbapenemase was KPC. These results suggest that CRE is an endemic disease despite the strengthened infection control policy of Gyeonggi-do; however, the isolation of CRE variants is increasing in long-term care hospitals and hospitals. Addressing this issue effectively requires the establishment of tailored infection control infrastructure in healthcare institutions and the development of specific government support measures.

Furthermore, to prevent the spread of healthcare-associated infectious diseases caused by multidrug-resistant bacteria, appropriate antibiotic usage should be implemented through antimicrobial stewardship programs [34,35], and active infection control measures—such as quarantine, contact testing, and monitoring—should be continuously performed by identifying strains at an early stage through prompt reporting. The reduction and prevention of the spread of CRE can be achieved through continuous and persistent infection control efforts by healthcare institutions, local governing bodies, and the government.

## 4. Methods

### 4.1. Data Collection

Data for this analysis were obtained through the integrated disease health management system of the KDCA (https://is.cdc.go.kr accessed on 1 August 2023). To ensure the security of surveillance data and protect personal information, identifying data other than demographic characteristics such as age or sex were discarded before analysis. The Gyeonggi-do hospital status data were obtained from the public data portal (https://www.data.go.kr accessed on 1 August 2023) used by the Ministry of Public Administration and Security. Moreover, healthcare facilities were classified into tertiary general hospitals and general hospitals and were analyzed using the current state data of Gyeonggi-do general hospitals, released by the Gyeonggi-do Health and Medical Policy Division on the website of the Gyeonggi-do Office (https://www.gg.go.kr accessed on 1 August 2023).

### 4.2. Statistical Analysis

This study involved a retrospective review of patient and death report data from cases in which CRE strains were isolated and reported in Gyeonggi-do over a 4-year period from 2018 to 2021. General characteristics, including the distribution of incidence by sex, age, and region, as well as overall incidence trends, were examined. Further, to determine the relationship between survival and death in relation to underlying disease and route of infection, as well as the major risk factors for healthcare-associated infection—including invasive procedures and history of hospitalization in an ICU—a chi-square test, an independent *t*-test, and Fisher’s exact test were conducted. Logistic regression analysis was used to analyze the ORs for correlation comparisons, with statistical significance set at *p* < 0.05. R version 4.2.1 and IBM SPSS version 26 were used for data analysis, and the analysis results were visualized using Tableau version 2020.4.

### 4.3. Definition of Cases

According to the 2022 healthcare-associated infectious disease management guidelines published by the KDCA, resistance to carbapenem antibiotics was defined as resistance to at least one carbapenem antibiotic—such as doripenem, imipenem, meropenem, or ertapenem—as evidenced in a culture test [34,35]. CPE detection was defined as the identification of CPE species through polymerase chain reaction among the strains classified as CRE.

In this study, the term “CRE-related survivors” was defined as patients with confirmed CRE-positive results in blood samples, who were alive at the time of the infectious disease notification. The term “CRE-related non-survivors” was defined as patients showing the presence of CRE, regardless of the presence of clinical symptoms, who died within 30 days after the last positive blood sample was collected. “Hospitalization” was defined as hospitalization history at the time of the CRE infection, and “antibiotics use” was defined as the antibiotic used by the patient during hospitalization. The test criteria for diagnosis included the isolation and identification of carbapenem-type antibiotic-resistant *Enterobacteriaceae* species from clinical samples through culture tests, after which the association between patient report history and pathogen report (culture test result) was also confirmed.

## Figures and Tables

**Table 1:** Demographical and clinical characteristics of patients with carbapenem-resistant *Enterobacteriaceae* bacteremia, 2018–2021.

	Survivors (*n* = 397)	Non-Survivors (*n* = 57)	t or χ²	*p*-Value
	N (%)	N (%)		
**Demographics**				
Sex, Female (%)	181 (45.6)	22 (38.6)	0.9867	0.3206
Age, median, years (range)	71 (0–96)	72 (50–97)	0.0382	0.5152
Age group, years				
0–9	2 (0.5)	0 (0.0)		
10–19	1 (0.3)	0 (0.0)		
20–29	7 (1.8)	0 (0.0)		
30–39	7 (1.8)	3 (5.3)		
40–49	20 (5.0)	5 (8.8)		
50–59	57 (14.4)	9 (15.8)		
60–69	93 (23.4)	11 (19.3)		
70–79	102 (25.7)	12 (21.1)		
80–89	94 (23.7)	12 (21.1)		
90–99	14 (3.5)	5 (8.8)		
**Clinical Characteristics**				
Hospitalization	366 (92.2)	46 (80.7)	7.8377	0.0051
Intensive care unit history	101 (25.4)	15 (26.3)		
Visit clinics	18 (4.5)	4 (7.0)		
Healthcare facilities *				
Tertiary hospitals	146 (36.8)	27 (47.4)		
General hospitals	211 (53.1)	25 (43.9)		
Long-term care facilities	32 (8.1)	4 (7.0)		
Hospitals	8 (2.0)	1 (1.7)		
**Antibiotic use** †				
Yes	161 (40.6)	17 (29.8)	0.9635	0.3263
Carbapenems	92 (46.9)	14 (49.1)		
Cephalosporins	87 (53.1)	6 (50.9)		
Fluoroquinolones	50 (35.5)	5 (32.1)		
Aminoglycosides	38 (26.9)	2 (7.1)		
Glycopeptides	43 (15.1)	6 (14.3)		
Other antibiotics	54 (29.0)	0 (0)		

* Healthcare facilities are classified as follows. Tertiary Hospitals: advanced medical centers, often associated with universities, that provide complex treatments for severe and unusual health conditions. General Hospitals: large healthcare institutions, typically having over 100 beds, providing a wide range of medical services across various specialties. Long-Term Care Facilities: institutions specifically designed for individuals who require continuous medical care and assistance with daily activities over extended periods. Hospitals: medical facilities, generally with over 30 beds, providing basic patient treatment with specialized staff and equipment. † Multiple selection.

**Table 2:** Underlying conditions of carbapenemase-producing *Enterobacteriaceae* bacteremia, 2018–2021.

	Survivors (*n* = 186)	Non-Survivors (*n* = 28)	t or χ²	*p*-Value	OR (95% CI)
**Underlying conditions**	**N (%)**	**N (%)**			
Diabetes	66 (35.5)	9 (32.1)	0.119	0.730	0.257 (0.422–3.743)
Hypertension	50 (26.9)	2 (7.1)	5.155	0.023	4.830 (1.045–22.328)
Brain stroke	28 (15.1)	4 (14.3)		1	1.329 (0.373–4.738)
Any malignancy	54 (29.0)	16 (57.1)	8.737	0.003	0.425 (0.183–0.990)
Renal failure	18 (9.7)	3 (10.7)		0.743	1.444 (0.290–7.177)
Liver disease	16 (8.6)	3 (10.7)		0.721	1.252 (0.280–5.590)
Chronic obstructive pulmonary disease	4 (2.2)	1 (3.6)		0.508	0.676 (0.052–8.795)
Dialysis	8 (4.3)	3 (10.7)		0.161	0.358 (0.055–2.319)
Immunosuppressed patients (immunosuppressant administration)	1 (0.5)	0 (0.0)		1	-
More than two underlying diseases	61 (32.8)	14 (50.0)	3.164	0.075	0.440 (0.188–1.033)
Other diseases	83 (44.6)	11 (39.3)	0.282	0.596	1.039 (0.380–2.842)

Abbreviations: OR, odds ratio; CI, confidence interval.

**Table 3:** Strains of carbapenem-resistant *Enterobacteriaceae* bacteremia, 2018–2021.

Strain	Survivors (*n* = 386)	Non-Survivors (*n* = 53)
	N (%)	N (%)
*Klebsiella pneumoniae*	230 (59.6)	37 (69.8)
*Escherichia coli*	89 (23.1)	13 (24.5)
*Enterobacter*	41 (10.6)	2 (3.8)
*Citrobacter freundii*	7 (1.8)	1 (1.9)
*Klebsiella oxytoca*	7 (1.8)	0 (0.0)
*Serratia marcescens*	7 (1.8)	1 (1.9)
*Citrobacter koseri*	2 (0.5)	1 (1.9)
*Providencia rettgeri*	3 (0.8)	0 (0.0)
Multiple strains	11 (2.8)	3 (5.7)
Others	6 (1.6)	1 (1.9)

**Table 4:** Carbapenemase-producing *Enterobacteriaceae* bacteremia, 2018–2021.

Strain *	Survivors (*n* = 212)	Non-Survivors (*n* = 29)
	N (%)	N (%)
KPC	151 (71.2)	25 (86.2)
NDM	49 (23.1)	5 (17.2)
OXA-48	11 (5.2)	0 (0.0)
VIM	0 (0.0)	0 (0.0)
IMP	0 (0.0)	0 (0.0)
GES	7 (3.3)	0 (0.0)

* Multiple selection KPC, *Klebsiella pneumoniae* carbapenemase; NDM, New Delhi metallo-β-lactamase; OXA-48, Oxacillinase-48; IMP, Imipenemase; VIM, Verona integron-encoded metallo-β-lactamase; GES, Guiana extended spectrum β-lactamase.

## Data Availability

The data used in this study are protected under the Personal Information Protection Act.
